# IS IT POSSIBLE TO OPTIMIZE STAPLED HEMORRHOIDOPEXY OUTCOMES BY ENLARGING OPERATIVE CRITERIA INDICATIONS? RESULTS OF A TAILORED PROCEDURE WITH ASSOCIATED RESECTION IN A COMPARATIVE PERSONAL SERIES

**DOI:** 10.1590/0102-672020220002e1696

**Published:** 2022-11-25

**Authors:** Fabio Guilherme Campos, Daiane Cavalari-Mancuzo, Leonardo Alfonso Bustamante-Lopez, Paula Gabriela Melo Morais, Carlos Augusto Real Martinez

**Affiliations:** 1 Universidade de São Paulo, Gastroenterology – São Paulo (SP), Brazil;; 2 Universidade Municipal de São Caetano do Sul, Gastroenterology – São Caetano do Sul (SP), Brazil;; 3 Universidade São Francisco, Gastroenterology – Bragança Paulista (SP), Brazil.

**Keywords:** Hemorrhoids, Hemorrhoidectomy, Recurrence, Rectal Prolapse, Treatment Failure, Hemorroidas, Hemorroidectomia, Recidiva, Prolapso Retal, Falha de Tratamento

## Abstract

**BACKGROUND:**

Since its introduction, stapled hemorrhoidopexy has been increasingly indicated in the management of hemorrhoidal disease.

**AIM:**

Our primary end point was to evaluate the incidence of recurrent disease requiring another surgical intervention. On a secondary analysis, we also compared pain, complications, and patient's satisfaction after a tailored surgery.

**METHODS:**

We retrospectively reviewed 196 patients (103 males and 93 females) with a median age of 47.9 years (range, 17–78) who were undergoing stapled hemorrhoidopexy alone (STG; n=65) or combined surgery (CSG; n=131, stapled hemorrhoidopexy associated with resection).

**RESULTS:**

Complications were detected in 11 (5.6%) patients (4.6% for STG vs. 6.1% for CSG; p=0.95). At the same time, symptoms recurrence (13.8% vs. 8.4%; p=034), reoperation rate for complications (3.1% vs. 3.0%; p=1.0), and reoperation rate for recurrence (6.1% vs. 4.6%; p=1.0) were not different among groups. Grade IV patients were more commonly managed with simultaneous stapling and resection (63% vs. 49.5%), but none of them presented symptoms recurrence nor need reoperation due to recurrence. Median pain score during the first week was higher in CSG patients (0.8 vs. 1.7). After a follow-up of 24.9 months, satisfaction scores were similar (8.6; p=0.8).

**CONCLUSION:**

Recurrent symptoms were observed in 10% of patients, requiring surgery in approximately half of them. Even though the association of techniques may raise pain scores, a tailored approach based on amplified indication criteria and combined techniques seems to be an effective and safe alternative, with decreased relapse rates in patients suffering from more advanced hemorrhoidal disease. Satisfaction scores after hemorrhoidopexy are high.

## RESUMO

**RACIONAL:**

Desde sua introdução, a hemorroidopexia por grampeamento tem sido cada vez mais indicada no manuseio da doença hemorroidária.

**OBJETIVOS:**

Nosso objetivo primário foi avaliar a incidência de doença recidivada que requeira tratamento cirúrgico. Numa análise secundária, também comparamos dor, complicações e satisfação do paciente após uma operação ajustada a cada caso.

**MÉTODOS:**

Foram revistos retrospectivamente 196 pacientes (103 homens e 93 mulheres) com idade média de 47,9 anos (17–78) submetidos a hemorroidopexia mecânica isoladamente (STG; n=65) ou cirurgia combinada (CSG; n=131, hemorroidopexia por grampeamento com ressecção).

**RESULTADOS:**

Complicações foram detectadas 11 (5,6%) pacientes (4,6% para STG vs. 6,1% para CSG; p=0,95). Ao mesmo tempo, recidiva de sintomas (13,8% vs. 8,4%; p=034), reoperações por complicações (3,1% vs. 3,0%; p=1,0) ou por recidiva (6,1 vs. 4,6%; p=1,0) não foram diferentes entre os dois grupos. Pacientes com grau IV foram mais comumente manuseados com grampeamento e ressecção simultâneos (63% vs. 49,5%), mas nenhum deles apresentou recidiva ou necessitou reoperação. O escore médio de dor na primeira semana foi maior no grupo CSG (0,8 vs. 1,7). Após seguimento de 24,9 meses, os índices de satisfação foram similares (8,6; p=0,8).

**CONCLUSÕES:**

Sintomas de recidiva foram observados em 10%, requerendo cirurgia em aproximadamente metade dos doentes. Embora a associação de técnicas aumente os escores de dor, um procedimento sob medida baseado em critérios ampliados de indicação e técnicas combinadas parece ser uma alternativa efetiva e segura, com menor recidiva em pacientes portadores de doença hemorroidária mais avançada. Os escores de satisfação após hemorroidopexia são altos.

## INTRODUCTION

Hemorrhoidal disease (HD) is an extremely frequent anorectal condition in adults, with estimates around 4.4% of the population and peak incidence from 45 to 65 years of age. Physiopathology includes vascular alterations (capillary shunts), inflammation (chronic irritation), mechanical (chronic constipation, frequent bowel motion, obesity, supine position, pregnancy, physical exercises), degenerative (connective and muscular support tissue destruction), and hormonal (pregnancy) factors^16^.

Prevalence of symptoms is greater with age and among women. Episodes of thrombosis lead to pain, local discomfort, and skin tags formation, mainly in supine position, when seating, or after defecation. Gradually, an external thrombosed and edematous blue pile covered by anoderm may ulcerate and bleed. In other patients, internal pinkish piles may prolapse from the anal verge and cause bleeding and irritation. Although an initial conservative treatment (fiber supplementation, warm water baths, suppositories, and creams) may attenuate symptoms, a proportion of patients will require other forms of treatment^11^.

The opportunity to choose the best treatment depends on personal experience, clinical presentation, and patient features. While grades I and II patients may be managed with conservative measures (such as rubber band ligation), more advanced disease will probably need surgical treatment (by excisional and nonexcisional techniques).

Classical hemorrhoidectomy techniques are still considered the “state of the art” for HD management. Recent data showed that the closed technique (Ferguson's) is superior to the open hemorrhoidectomy operation (Milligan-Morgan) in terms of reducing postoperative bleeding or severe pain. Ferguson's technique was also proven to be associated with faster wound healing^2^.

However, the associated pain and slow recovery after excisional surgery led to the development of innovative nonexcisional procedures such as stapled hemorrhoidopexy (SH) and Doppler-guided hemorrhoidal dearterialization with mucopexy (DG-HAL) for grades III and IV patients^11^.

The concept of SH (also called stapled hemorrhoidectomy or mechanical anopexy) was introduced by Longo in 1998^12^ as an alternative for conventional techniques, shifting our attention to the rectal wall above the prolapsed hemorrhoids. The use of circular stapler allows to excise a circumferential strip of mucosa that reduces prolapse degree and pulls the hemorrhoidal cushions to their original anatomical position. Thus, the mucosectomy aims to obtain prolapse correction (rectopexy), reduce submucosal vascular supply to hemorrhoidal plexus, and preserve the anoderm. In conjunction, these modifications may control symptoms and restore anal canal function and anatomy.

Besides its advantages, higher recurrence rates and severe complications related to the stapling have been reported^8,10,13^. Throughout time, improvement of devices, close adhesion to technical details, and progressive experience have played a key role to achieve better outcomes. Within this context, the aim of this study was to present technical modifications and change of concepts we have developed overtime in this operative technique and to analyze postoperative outcomes after a tailored management according to clinical presentation.

## METHODS

A retrospective analysis of patients undergoing SH in a private setting was performed. Clinical and surgical data of those operated from 2010 to 2020 were retrieved from medical records containing prospectively registered data. Our primary end point was to evaluate the incidence of recurrent disease or symptoms requiring another surgical intervention. On a secondary analysis, we compared pain, complications, and satisfaction after procedures indicated selectively according to disease features.

Retrieved data included age, gender, symptoms (prolapse, bleeding, local pain, itching, burning), complaints of chronic constipation, family history of HD, previous operations, HD grade, type of operative procedure (SH or SH combined with resection), postoperative pain (pain scale), operative morbidity, complications related to reoperations, length of follow-up (months), symptoms of hemorrhoidal recurrence, and need for medical treatment or reoperations due to recurrent disease. A visual analog scale was used to evaluate postoperative pain at the end of the first week after surgery.

Data collection was complemented by sending a questionnaire to all patients and through telephone calls. A last evaluation was performed through a written questionnaire sent to all patients, in order to check their satisfaction. This was categorized as low (1–3), moderate (4–6), satisfied (7–8), and very satisfied (9–10).

### Surgical Technique

None of the patients were treated in an outpatient basis or with local anesthesia. Preoperative preparation included rectal washout and endovenous antibiotics 1 h before the procedure. Under sedation (to avoid unconscious movements) and spinal anesthesia, they were placed in lithotomy and Trendelenburg position. The internal anorectal prolapse was assessed through digital examination and with endoanal gauze introduction-retrieved movement. Prolapse was then reduced with the introduction of Circular Anal Dilator (CAD) via endoanal and positioning of the Purse-String Suture Anoscope.

A submucosal continuous suture using 2–0 Prolene was started at 3 o'clock position and progressed clockwise. At the left lateral position (9 o'clock), the Prolene suture was enlaced with another 2–0 nonabsorbable stitch and the suture progressed toward the point at the right lateral where it was started. At the end, we obtained 2 traction points situated at the right (3 o'clock) and left (9 o'clock). The head of the 33 mm circular stapler was then introduced beyond the suture to allow mucosal approximation around the stapler axis. After stapler closing and firing, it was opened and removed. The staple line and the mucosectomy specimen were checked for bleeding and integrity. The verification of dehiscence or bleeding at the staple line was immediately corrected with absorbable stiches involving the suture line.

Statistical analysis was performed employing the chi-square test and Yates correction, Fischer's exact test, and Kruskal-Wallis test. The study was approved by the Ethics Committee of the University Hospital of the Universidade de São Paulo (USP).

## RESULTS

During the study period, we identified 280 patients operated for HD. Operative procedures included SH (196; 74.8%), excisional hemorrhoidectomy (66; 23.5%), and DHAL (18; 6.4%). Among the 196 SH patients, we identified 65 (33.2%) treated only with mechanical anopexy (named SH group or SHG) and 131 (66.8%) who underwent mechanical anopexy complemented with hemorrhoids or skin tags resection (named combined surgery group or CSG). Combined procedure was indicated in cases presenting external disease on proctological examination or at the external evaluation after stapling.

The most common reported hemorrhoidal symptoms reported by the 196 SH patients are listed on Table 1. Intestinal constipation, family history of HD, and previous surgery were mentioned by 64 (32.5%), 35 (17.9%), and 11 (5.8%) patients, respectively.

**Table 1:** Symptoms related to hemorrhoidal disease in 196 patients undergoing stapled hemorrhoidopexy.

Symptom	Number	%
Prolapse	139	70.9
Bleeding	69	35.2
Anal pain	31	15.8
Thrombosis	27	13.8
Other	19	9.7

Patients undergoing SH comprised 103 (52.5%) males and 93 (47.4%) females, with ages varied from 17 to 78 years (median 47.9). These characteristics and other data regarding comparative results among SHG and CSG are summarized in Table 2. Most patients belonged to HD stage II (44; 22.4%) and stage III (129; 65.8%).

**Table 2:** Demographic data and operative results

Variables (entire series)		SHG (65)	CSG (131)	p-value
Sex ratio				
	*Male (103; 52.5%)*	*Male (43;66.2%)*	60 (45.8%)	0.011 *
	*Female (93; 47.4%)*	*Female (22; 33.8%)*	71 (54.2%)	
	Age (years) 47.9 (17 to 78)	49.8 (17–78)	47.3 (24–78)	0.147#
Hemorrhoidal				
	I (2; 1.0%)	2 (2.1%)	0	
	II (44; 22.4%)	13 (20.0%)	31(23.7%)	
	III (129; 65.8%)	47 (49.5%)		
	IV (21; 10.7%)	3 (4.6%)	82(62.6%) 18(13.7%)	
Pain score (variation)		0.8 (0–8)	1.7 (0–10)	0.0009#
Morbidity rates 11 (5.6%)		3 (4.6)	8 (6.1%)	0.95+
Recurrent symptoms 20 (10.2%)		9 (13.8%)	11 (8.4%)	0.34+
Reoperations for complications 6 (3.0%)		2 (3.1%)	4 (3.0%)	1.00+
for recurrence 10 (5.1%)		4 (6.1%)	6 (4.6%)	
Satisfaction score (variation)		8.6 (5–10)	8.6 (4–10)	0.81#
Follow-up (months) 24.9 (1–204)		20.3 (1–204)	24.4 (1–192)	0.72#

* qui-square and Yates correction; +Fischer Exact test;

# Kruskal-Wallis test; SHG: mechanical anopexy; CSG: mechanical anopexy complemented with hemorrhoids or skin tags resection.

Patients classified maximal pain intensity during the first postoperative week as 0.8 (0–8) in SHG and 1.7 (0–10) in CSG, respectively. Overall, 30 days morbidity was registered in 11 (5.6%) patients, being 3 (4.6%) in SHG and 8 (6.1%) in CSG. Operative complications are listed in Table 3. Persistent anal pain and/or tenesmus were classified as complications when it affected quality of life.

**Table 3:** Operative complications among 196 stapled hemorrhoidectomy.

Complications	Number	Clinical management	Surgical management
Partial stenosis	5 (2.5%)	2	3
Anal pain	3 (1.5%)	2	1
Abscess	1 (0.5%)	0	1
Thrombosis	2 (1.0%)	1	1
Total	11 (5.6%)	5 (45.4%)	6 (54.5%)

There was no statistical difference among both groups regarding morbidity, symptoms recurrence, and reoperations. Recurrent symptoms of HD requiring clinical management were registered in 20 (10.2%) patients [9 (13.8%) vs. 11 (8.4%)]. However, reoperations for recurrence were necessary in only 10 (5.1%) patients, with no difference among STG (4; 6.1%) and CSG (6; 4.6%). These reoperated patients were classified as stage II (3; 6.8%) and IV (7; 5.4%) diseases.

Reoperations due to postoperative complications were necessary in 6 (3.0%) of total patients (or 60% of those who presented complications), being 2 (3.5%) in SHG and 4 (2.9%) in CSG. These 6 patients were stage III (5/129; 3.9%) and stage IV (1/4.7%) diseases.

Our questionnaire was responded by 44 SHG and 99 CSG patients. After more than 20 months of follow-up, patients attributed similar median satisfaction score of 8.6 in SHG (50–10) and CSG (4–10). Satisfaction was considered complete (scale 8–10) for 36 patients in SHG (44 answers) and 86 (86.9%) patients among 99 who responded that answer in the questionnaire.

## DISCUSSION

Introduction of SH into clinical practice has provided better postoperative outcomes concerning postoperative comfort and recovery^18^. Symptoms control is attributed to interruption of blood supply, improvement of venous drainage, and anatomical repositioning. Within the period of the present study, SH comprised 75% of all surgical options.

When the technique was originally described, cases of the third and fourth degrees internal prolapses and patients exhibiting minor disease considered refractory to medical management were considered the ideal candidates. On the contrary, those presenting prolapsed cushions or fibrotic piles were preferably treated by resection techniques.

Overtime, indications criteria have been amplified, and factors related to anatomy (one or two prolapsed piles, external thrombosis), symptoms (bleeding, thrombosis), or associated diseases (obstructed defecation) were no longer considered obstacles to perform SH^19^.

It has been widely recognized that a meticulous observation of technical details is crucial to avoid complications and recurrence. Morbidity rates varying from 9 to 15% have been reported, which include bleeding, persistent rectal pain, urgency to defecate, partial stenosis, external thrombosis, suture dehiscence, and local submucosal abscess^18^. Although majority of symptoms are of minor importance, some rare life-threatening events may also occur. Gradually, the development of new devices turned bleeding a very rare occurrence. Furthermore, a meticulous inspection after firing may preclude the need for an easy manual suture with absorbable stiches to avoid local bleeding^19^.

As summarized in Tables 2 and 3, we registered complications in only 11 (5.6%) patients, with no difference among SHG (5.2%) and CSG (5.7%). Partial stenosis was diagnosed in 5 (2.5%) patients, but only 3 (1.5%) required surgical correction. This is the reason why we advocate digital rectal examination in the postoperative period, aiming to detect and avoid the development of anal stenosis, especially when the final suture line remains too close to the dentate line. In these cases, pain and tenesmus may be lessened by repeated digital rectal examinations, surgical plasty of anorectal stricture, or even scar tissue removal. This complication may be surgically managed with low morbidity and high efficacy, mainly when revisional surgery is performed within 3 months after surgery^18^.

As a matter of fact, the purse-string suture must not be too high (to provide an adequate prolapse retraction), too deep (to avoid muscular inclusion), or too low (so the stapler line could involve the dentate line and cause pain). Most commonly, the circular manual suture is fashioned 2.5–3.5 cm proximally to the dentate line^5^. Besides, 3 (1.5%) of our patients had a complaint of important anal pain. As the extent of the transitional epithelium may vary individually, some cases may experience occasional discomfort. Also, the eventual presence of muscular fibers in the surgical specimen has not been associated with rectal pain or functional disturbances.

We do believe that the correct application of an operative technique plays a major role in its recurrence rates. Literature reviews have demonstrated greater recurrence rates after SH when compared to excisional techniques. Some controversy exists among those who believe these higher rates are due to improper inclusion of residual skin tags into the recurrence data^8^.

Consequently, SH must be offered to patients after information of these data and confirmation of surgeon's experience. In our series, only 10.2% of patients referred recurrent symptoms during follow-up, with a not statistical difference among both surgical options (13.8% for SHG vs. 8.4% for CSG), similarly to other series^1^. In a group of 257 patients followed more than 10 years, recurrence has been reported in up to 47% of patients, although reintervention was necessary in only 15%^17^.

Among our patients, half (5.1%) of those presenting symptoms required a subsequent reoperation, with no different rates between the two groups. When we add all reoperations due to complications and recurrences (total 16 cases, 8.2%), both SHG (6; 6.3%) and CSG (10; 7.6%) seemed to provide similar outcomes in this setting. If we think that a combined surgery was offered to patients probably presenting a more advanced disease (exhibiting external thrombosis, skin tags, or refractory prolapse even after firing the stapler), we should expect higher rates of recurrence and reoperations among this specific group. Fortunately, we were probably capable of preventing this unfortunate evolution by adding an additional resection to the mucosectomy with stapling.

Certainly, such a heterogeneous disease should not receive a standardized management for all cases. Previous reports have already suggested that, in selected cases, different strategies could be employed in the presence of inelastic internal piles, external disease, or skin tag not tolerated by the patient^4,6,7,14^. Consequently, a more effective disease control may be accomplished by performing additional or combined procedures, even if they cause pain. The present study raises the importance of complementing SH by adding procedures such as limited resections of internal and external thrombosed hemorrhoids or skin tags, aiming to improve long-term outcomes.

As expected, grade III patients manifested recurrence of symptoms more frequently than grade II (Table 3), even though with similar index of reoperations. Among our 196 patients, grade IV patients were more commonly managed with simultaneous stapling and resection (63% vs. 49.5%). As summarized in Table 3, none of those classified as grade IV presented symptoms recurrence nor they need reoperation due to recurrence (Table 4).

**Table 4:** Hemorrhoidal disease distribution and results regarding symptoms recurrence and reoperations.

Hemorrhoidal grades (n=196)	SHG (65)	CSG (131)	Recurrence of symptoms*	Reoperations for recurrence*
I (2; 1.0%)	2 (1.0%)	0	0	0
II (44; 22.4%)	13 (20.0%)	31 (23.7%)	2 (4.5%)	3 (6.8%)
III (129; 65.8%)	47 (49.5%)	82 (62.6%)	18 (13.9%)	7 (5.4%)
IV (21; 10.7%)	3 (4.6%)	18 (13.7%)	0	0

* p=0.56 Kruskal-Wallis test; SHG: stapled hemorrhoidopexy group; CSG: combined surgery group.

In the literature, greater recurrence rates and persistence of HD currently observed after treating grade IV patients may suggest that the device could be insufficient to adequately resect a great extension (or volume) of internal prolapse^7^. Our results in this group of patients suggest that the association of two procedures in the same patient may provide a more effective disease control.

A total of 73% of the questionnaires were sent back, allowing us to verify a high score of patients’ satisfaction (8.6), meaning they were very happy with treatment. In Table 5, similar impressions reported by others are presented.

**Table 5:** Patient satisfaction in literature series.

Authors	n	Satisfied (%) or very satisfied scores
Araujo, 2016	86	84
Sturiale, 2018	171	81
Schneider, 2019	257	63
Puia, 2021	35	94
Present series	143	8.6

Based on the concept that internal rectal prolapse participates in the disease process, the current experience allows us to consider SH as a major innovative and revolutionary advance in HD treatment. Similarly, dearterialization technical options (Doppler and non-Doppler-guided, tailored mucosectomy) also deal with the same problem. These interesting alternatives should deserve correct indication and proper technical execution by experienced surgeons aware of technical details and potential morbidity.

Currently, comparison of these two techniques still leads to different conclusions. In a recent comparative study^9^, early and late results of SH (50) and HD (100) for grades III and IV after 2 years of follow-up showed greater recurrence rates (16% vs. 4%), pain scores, operative length, and recovery period for HD. These results turn general adoption of dearterialization a difficult task^3,15^. A meta-analysis that reviewed six randomized trials comparing 274 SH and 280 HD demonstrated greater recurrence rates (13.2% vs. 6.9%) for HD, whereas complications (17.1% vs. 23.3%) and patients’ satisfaction were similar in both groups^7^.

## CONCLUSION

The comparative results observed in the present study suggest that improved outcomes after SH may be achieved when indications criteria include those diagnosed with larger piles, external thrombosis, and skin tags, by performing additional limited resection. Thus, a tailored approach to HD seems to be an effective alternative aiming to decrease relapse in cases suffering from more advanced disease, even though the combination of techniques demonstrated to be associated with greater pain in this group (1.7 vs. 0.8).

Moreover, although we do not have comparative results to analyze, the technical modification characterized by the presence of two opposite traction points in the submucosal circumferential suture seems to lead a greater mucosectomy. This optimized mucosectomy probably contributed to the excellent long-term results we have observed among our patients.
